# AgroBench and ARI: A reproducible method for robustness evaluation of agricultural object detectors

**DOI:** 10.1016/j.mex.2026.104068

**Published:** 2026-07-25

**Authors:** Yassine Zarrouk, Abdelhak Khallou, Mohammed Bourhaleb, Mohammed Rahmoune, Khalid Hachami

**Affiliations:** a LaRSA, ENSAO, Mohammed First University, Oujda, Morocco; b SMART, SupMti, Oujda, Morocco; c National Institute of Agronomic Research, Regional Centre of Agricultural Research, Oujda, Morocco

**Keywords:** Agricultural object detection, Robustness evaluation, Corruption benchmark, Reproducible method, AgroBench, Agro-Robustness Index, Model validation

## Abstract

Robustness evaluation of agricultural object detectors is often limited to clean-set metrics, which can conceal substantial performance degradation under realistic field perturbations such as haze, motion blur, compression artifacts, chromatic shifts, and canopy-related occlusions. This article presents a reproducible method that combines an agriculture-calibrated corruption benchmark, termed AgroBench, with a normalized robustness summary metric, the Agro-Robustness Index (ARI). The method is designed to quantify how much of a detector’s clean performance is retained under a fixed, domain-grounded corruption protocol while preserving per-family diagnostic interpretability. AgroBench specifies explicit corruption families, parameter grids, and global modulation settings to ensure deterministic and comparable evaluation across runs. ARI summarizes corrupted performance as a normalized retention score relative to each detector’s clean reference, thereby reducing the influence of baseline clean accuracy on robustness interpretation. The method is demonstrated on agricultural object detection using YOLOv8n and produces both a global robustness score and per-family retention components that support diagnosis and reporting. This framework is intended as a reusable evaluation method for standardized robustness assessment in agricultural vision.

## Graphical abstract

**Figure:**
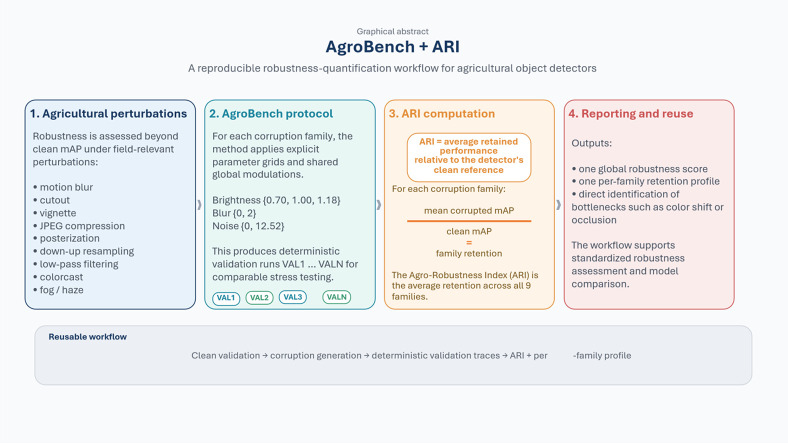

## Specifications table

**Table:** 

**Subject area**	Agricultural and Biological Sciences
**More specific subject area**	*Agricultural computer vision; object detection robustness evaluation; corruption-based benchmarking for plant disease detection*
**Name of your method**	*AgroBench and ARI: an agriculture-calibrated corruption benchmarking and robustness evaluation method for object detectors*
**Name and reference of original method**	*This method customizes corruption-based robustness evaluation for computer vision and adapts it to agricultural object detection. A key methodological precursor is: Kamann, C., Rother, C., Benchmarking the Robustness of Semantic Segmentation Models with Respect to Common Corruptions* [1]. *The agriculture-calibrated corruption protocol, benchmark configuration, and Agro-Robustness Index introduced here are original to this work.*
**Resource availability**	*The camera-ready software release used to produce the results in this manuscript is archived under the Zenodo version DOI****10.5281/zenodo.18910668****(release****v1.0.1****). The Zenodo concept DOI****10.5281/zenodo.18909108****resolves to the latest available release. The released materials include corruption-generation scripts, evaluation scripts, benchmark configuration files, exported validation traces, environment specifications, and example commands for reproducing the benchmark and ARI computation. The method requires an annotated agricultural object detection dataset, a trained detector, Python, OpenCV, PyTorch, and the Ultralytics validation framework.*

## Background

*Deep learning-based object detection is increasingly used in agricultural vision for tasks such as plant disease monitoring, weed detection, and field scouting. In practice, these systems are often evaluated primarily on clean validation sets, where performance is typically summarized by precision, recall, and mean Average Precision. Although recent corruption-based robustness benchmarks have highlighted the importance of evaluating models beyond clean-set performance* [1,2], *these general-purpose approaches are not tailored to the characteristics and deployment conditions of agricultural vision systems. While clean-set metrics remain essential, they do not directly quantify how well a detector retains its performance when exposed to realistic image degradations encountered during deployment. In agricultural conditions, such degradations are common and include haze, motion blur, compression artifacts, chromatic shifts, sensor noise, and partial occlusions caused by leaves or canopy structure. As a result, a detector may appear highly reliable on clean images while remaining fragile in real operating environments.*

*This methodological gap is important for agricultural applications because deployment conditions are rarely controlled. Field acquisition varies with weather, illumination, optics, platform movement, and transmission constraints. Previous work in this research line also showed that changes in preprocessing and image conditions can substantially affect model behavior, including generalization, latency, and detection accuracy in agricultural settings.*

*The method presented here was developed to provide a reproducible way to evaluate detector robustness beyond clean performance. It combines an agriculture-calibrated corruption benchmark, AgroBench, with a normalized robustness summary metric, the Agro-Robustness Index (ARI). AgroBench defines a fixed set of corruption families, explicit parameter grids, and deterministic global modulation settings so that corrupted evaluations can be reproduced across runs and reused across datasets. ARI summarizes corrupted performance as retention relative to the detector’s own clean reference, while also preserving per-family outputs for diagnosis. This allows robustness to be interpreted as a complementary evaluation axis rather than as a replacement for clean accuracy.*

*This methodology may be used whenever researchers need to profile the robustness of agricultural object detectors under realistic inference-time perturbations, compare detectors under a shared corruption protocol, or identify dominant failure modes before deployment. In its current form, the method is demonstrated on YOLOv8n as a robustness profiling workflow, with cross-model ranking left for future extensions.*

## Method details

### Overview of the AgroBench and ARI workflow

The proposed method provides a reproducible workflow for evaluating the robustness of agricultural object detectors under realistic inference-time image degradations. It combines two complementary components: AgroBench, an agriculture-calibrated corruption benchmark, and ARI, the Agro-Robustness Index. AgroBench defines the corruption protocol used to generate controlled degraded versions of a clean validation set, whereas ARI summarizes the detector’s retained performance under that protocol relative to its clean reference.

The workflow begins with a trained object detector and an annotated clean validation set. A clean evaluation is first performed to obtain the detector’s baseline performance under fixed validation settings. This clean score serves as the reference for later normalization. The clean validation images are then transformed using the AgroBench corruption protocol, which applies predefined corruption families, explicit family-specific parameter grids, and fixed global modulation settings. For each corruption configuration, a corrupted validation subset is generated while preserving the original annotations.

**Fig. 1:**
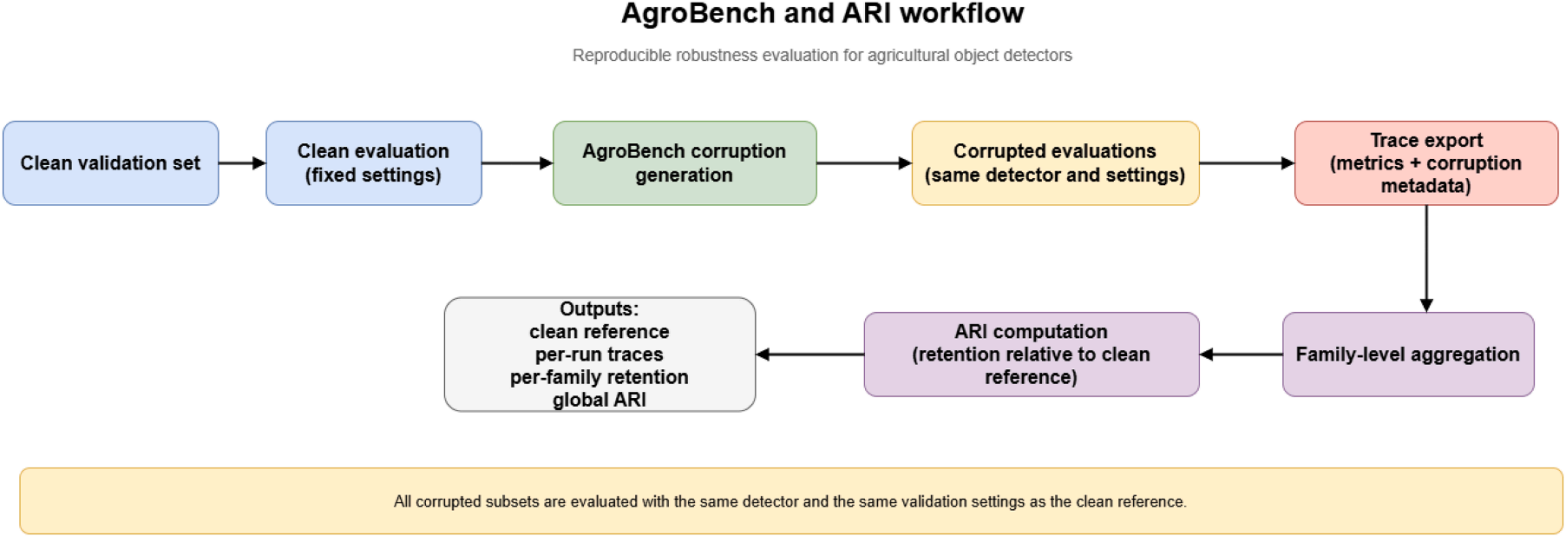
AgroBench and Agro-Robustness Index (ARI) evaluation pipeline. A clean validation set establishes the baseline performance, after which AgroBench generates agriculture-calibrated corrupted subsets for evaluation under identical settings. Evaluation traces are aggregated to compute the normalized ARI and per-family robustness indicators.

Each corrupted subset is evaluated using the same detector and the same validation settings as the clean reference. The resulting outputs are exported as structured evaluation traces that record corruption metadata together with detection metrics. These traces are then aggregated in two stages. First, corrupted performance is averaged within each corruption family over all predefined parameter settings and global modulation tuples. Second, each family-level average is normalized by the detector’s clean reference to compute a family-specific retention ratio. The global ARI score is obtained by averaging these retention ratios across all corruption families Fig. 1.

The method therefore produces four complementary outputs: the clean reference performance, the per-run corrupted evaluation traces, the per-family retention components, and the global ARI score. Together, these outputs enable both reproducible robustness reporting and diagnostic analysis of corruption-specific failure modes in agricultural vision systems.

### Input requirements

The AgroBench and ARI workflow requires a trained object detector, an annotated clean evaluation set, and a software environment capable of generating corrupted image variants and running detector validation under fixed settings. The method is intended for agricultural object detection, but it can be adapted to other vision domains if the corruption families and calibration rationale are redefined.

The primary input is an annotated clean validation set that represents the reference distribution $D_{0}$. Images should be stored in a standard raster format such as PNG or JPEG, and annotations should remain available in the detector’s expected format. In the present implementation, labels are provided in YOLO format and are copied unchanged to each corrupted validation subset because the method assumes that corruptions alter image appearance without changing object identity or geometry. The clean validation set must be fixed before corruption generation because it serves as the common reference for all subsequent evaluations.

The second required input is a trained detector. The method does not require a specific architecture, but the model must support deterministic evaluation on both clean and corrupted subsets using the same validation configuration. In the demonstration described in this work, YOLOv8n is used as the reference detector and evaluated through the Ultralytics validation interface. The same workflow can be reused with other detectors provided that clean and corrupted runs are executed under identical settings and that the base metric remains consistent throughout ARI computation.

The third requirement is a software environment for corruption generation and validation. In the released implementation, the method depends on Python, OpenCV, PyTorch, and the Ultralytics framework. The corruption-generation and evaluation scripts are distributed with the archived software release linked in the Specifications table. To reproduce the method, users must have access to the benchmark scripts, the environment specification files, and the configuration files defining corruption families, parameter grids, and global modulation settings.

A minimal hardware requirement is the availability of a system capable of running detector validation on the selected model and dataset size. GPU acceleration is recommended for practical execution time, but it is not a conceptual requirement of the method. The demonstration reported in this article uses a GPU-enabled environment, although the workflow itself remains independent of a specific hardware platform.

For clarity and reproducibility, it is recommended to summarize these requirements in a dedicated table in the manuscript Table 1.

**Table 1:** Input requirements and software resources required to reproduce the AgroBench and Agro-Robustness Index (ARI) evaluation workflow, together with the corresponding implementation used in this study.

Component	Requirement	Example used in this study
**Clean evaluation dataset**	A fixed annotated clean validation set representing the reference distribution used for baseline evaluation and corruption generation	Potato late blight validation split derived from a 14,150-image agricultural dataset
**Image files**	Standard raster image format readable by the corruption-generation pipeline	RGB agricultural images stored as PNG or JPEG
**Annotation format**	Object detection annotations must be available in a format compatible with the selected detector; annotations remain unchanged across corrupted subsets	YOLO annotation format
**Trained detector**	A trained object detector that can be evaluated deterministically on both clean and corrupted subsets using identical validation settings	YOLOv8n
**Base detection metric**	A fixed detection metric used consistently for clean and corrupted evaluation and for ARI computation	mAP@50
**Corruption-generation software**	Scripts or software capable of applying the AgroBench corruption families, family-specific parameter grids, and global modulation tuples to the clean validation images	Released *prepare_valset.py* implementation
**Validation framework**	A detector evaluation interface that can validate the selected model on the clean and generated corrupted subsets under fixed settings	Ultralytics YOLO validation
**Software environment**	Python environment with the libraries required for corruption generation, model loading, and validation	Python, OpenCV, PyTorch, Ultralytics
**Archived reproducibility resources**	Publicly accessible release containing the camera-ready scripts, benchmark settings, environment files, and example execution commands	Zenodo version DOI: ***10.5281/zenodo.18910668***; concept DOI: ***10.5281/zenodo.18909108***
**Hardware used in demonstration**	System capable of running detector validation on the selected dataset and model; GPU acceleration is recommended for practical runtime	GPU-enabled environment used for the YOLOv8n demonstration
**Output storage**	File system space for corrupted validation subsets, exported validation traces, and ARI aggregation outputs	One CSV trace per corruption family

### Construction of the agriculture-calibrated corruption benchmark

The agriculture-calibrated corruption benchmark, termed AgroBench, was designed to evaluate agricultural object detectors under realistic inference-time image degradations while preserving deterministic and reproducible execution. The benchmark operates on a fixed clean validation set and generates corrupted evaluation subsets by combining one corruption family, one predefined family-specific parameter setting, and one fixed global modulation tuple. Each resulting subset is then evaluated under the same validation settings used for the clean reference. This design ensures that robustness differences can be attributed to controlled perturbations rather than to changes in annotations, evaluation settings, or stochastic generation procedures.

AgroBench was constructed to reflect recurrent agricultural acquisition effects that are not captured by clean-set reporting alone. The benchmark therefore emphasizes field-relevant perturbations such as texture loss, resolution degradation, platform-induced motion, haze-like attenuation, compression artifacts, canopy occlusion, low-cost optics effects, quantization artifacts, and chromatic drift. In contrast to generic corruption suites, the benchmark uses explicit parameter grids calibrated as reproducible defaults for agricultural imagery and preserves a fixed composition rule across all evaluations.

#### Corruption families and agricultural rationale

Let Fdenote the set of corruption families used in AgroBench. Each family is motivated by a plausible field acquisition cause and implemented as a parameterized transformation operator $T_{f,s}$, where fidentifies the corruption family and sdenotes one family-specific parameter setting. In the current release, AgroBench includes nine corruption families: **Lowpass, Downup, Motion blur, Fog, JPEG, Cutout, Vignette, Posterize, and Colorcast**. These families were selected to approximate realistic perturbations encountered during agricultural deployment, including defocus and loss of fine texture, resolution loss due to resizing or transmission, vibration-induced motion blur, haze or dust attenuation, lossy compression, partial visibility due to canopy clutter, lens shading, reduced bit-depth artifacts, and white-balance or illumination-spectrum drift. Each evaluation sample contains only one family corruption, which is subsequently combined with the shared global modulation procedure described below.

#### Family-specific parameter grids

**Fig. 2:**
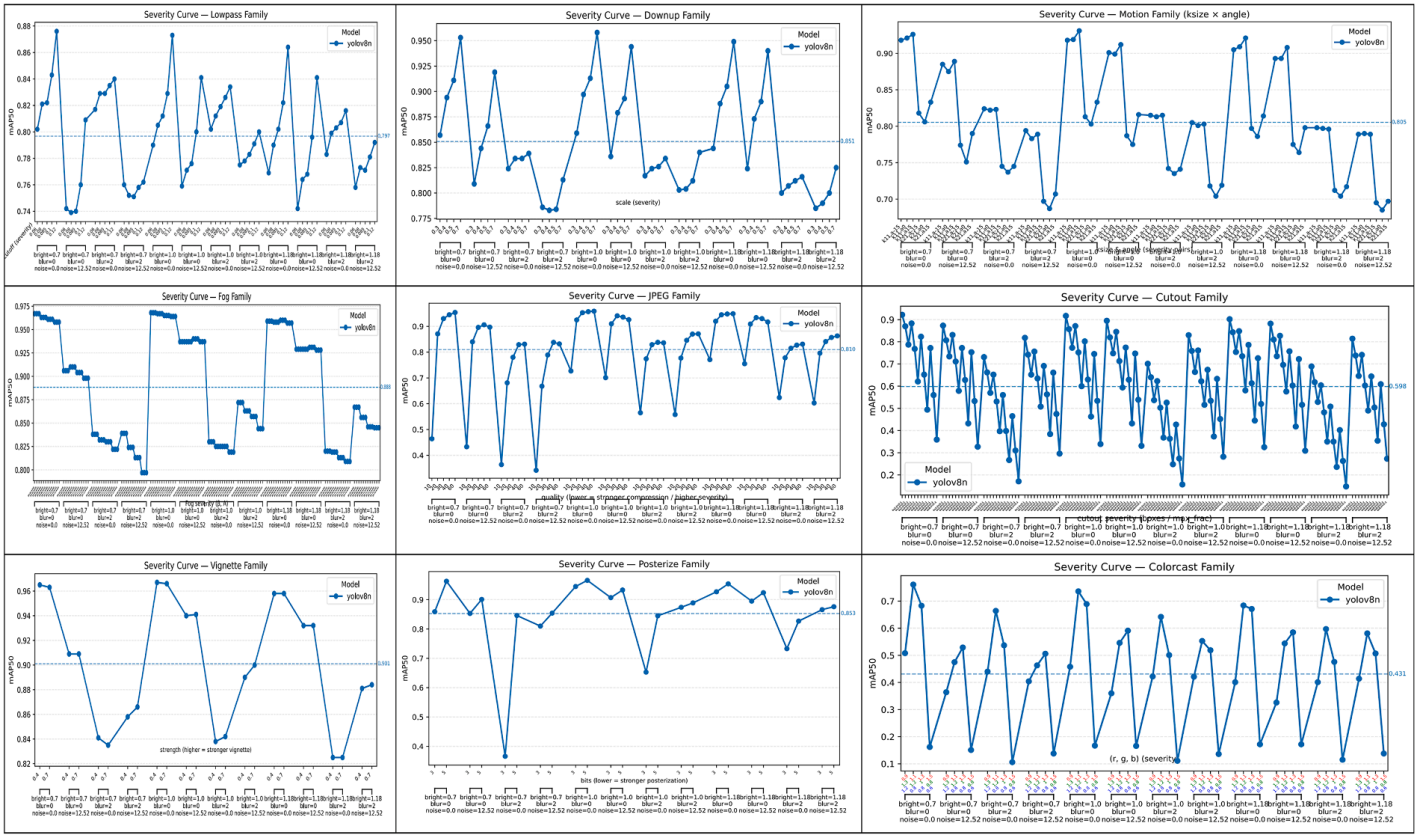
Representative severity curves for the corruption families implemented in AgroBench. Each curve illustrates the progression of detector performance across the predefined parameter settings and global modulation combinations for a given corruption family.

For each corruption family f∈F, AgroBench defines an explicit discrete parameter grid $S_{f}$. The number of settings may differ across families because the implemented operators have different parameterizations. Severity grids were chosen according to three calibration principles: **field plausibility, monotonic difficulty**, and **cross-family comparability**. Field plausibility requires the selected parameter ranges to remain visually realistic for agricultural acquisition. Monotonic difficulty requires increasing parameter strength to produce progressively stronger distortion in most cases. Cross-family comparability requires each family grid to represent a meaningful progression of corruption strength while acknowledging that exact perceptual equivalence across families is not guaranteed. These grids are distributed as part of the benchmark specification and can be refined for other datasets or sensors if the calibration rationale is reported explicitly Table 2. Representative severity curves illustrating the effect of the implemented parameter grids across the AgroBench corruption families are shown in Fig. 2.

**Table 2:** Summary of the AgroBench corruption families. For each family, the table lists the targeted agricultural acquisition cause, the implemented image transformation operator, and the associated severity parameter grid used to generate corrupted validation subsets.

Family (f)	Field acquisition cause	Operator (T_f,s)	Implemented severity grid (S_f)
Lowpass	Loss of fine texture (defocus, optics blur)	FFT low-pass filtering	cutoff ∈ {0.06, 0.08, 0.085, 0.10, 0.12}
Downup	Resolution degradation (distance, resizing, transmission)	Down-sample then up-sample	scale ∈ {0.70, 0.50, 0.40, 0.30}
Motion	Platform vibration, camera shake, wind-induced motion	Linear motion blur	ksize ∈ {11, 21}, angle ∈ {−15, 0, 15}
Fog	Haze, dust, early-morning attenuation, spray drift	Atmospheric scattering model	β ∈ {0.05, 0.08, 0.11, 0.14}, A ∈ {200, 220, 240}
JPEG	Bandwidth/storage constrained pipelines	JPEG re-encoding	quality ∈ {60, 40, 30, 20, 10}
Cutout	Canopy clutter and partial visibility (leaf/branch occlusion)	Structured occlusion masks	boxes ∈ {2, 4, 6, 8}, max_frac ∈ {0.10, 0.20, 0.30}
Vignette	Low-cost optics, lens shading, edge darkening	Radial attenuation	strength ∈ {0.40, 0.70}
Posterize	Quantization / reduced bit depth imaging	Bit-depth reduction	bits ∈ {5, 3}
Colorcast	White-balance drift, illumination spectrum variation	Channel scaling (r, g, b)	(r, g, b) ∈ {(1.5, 0.8, 0.6), (1.3, 1.0, 0.8), (1.1, 1.2, 0.8), (0.9, 1.3, 1.2)}

#### Global modulation settings

In addition to the primary corruption operator $T_{f,s}$, AgroBench applies a set of global modulations that are shared across all corruption families. These modulations emulate low-amplitude fluctuations of the imaging chain while preserving comparable test conditions across families. In the current release, the global modulation tuple is written as:

$$b=\left(b_{\text{bright}},b_{\text{blur}},b_{\text{noise}}\right)$$

where the three components correspond respectively to brightness scaling, Gaussian blur, and additive Gaussian noise.

The discrete grids are:

$$b_{\text{bright}}\in \left\{0.70,1.00,1.18\right\}$$

$$b_{\text{blur}}\in \left\{0,2\right\}$$

$$b_{\text{noise}}\in \left\{0,12.52\right\}$$

This yields:

3×2×2=12

global modulation tuples for each family-specific corruption setting. These values were selected to create both mildly altered and more challenging conditions while remaining visually plausible for agricultural imagery.

At the run level, one global modulation tuple is fixed for all images belonging to the generated corrupted subset. The benchmark therefore enumerates the Cartesian product of the three global modulation grids and combines each tuple with each family-specific parameter setting. This produces a deterministic set of corrupted evaluation subsets that can be regenerated identically across runs.

#### Corruption composition rule

Each corrupted image is produced by composing the global modulation operator with one family-specific corruption operator according to:

$$I_{f,s,b}=T_{f,s}\left(G_{b}\left(I\right)\right)$$

where Iis the clean image, $G_{b}$denotes the global modulation defined by the selected tuple b, and $T_{f,s}$denotes the family-specific corruption.

**Fig. 3:**
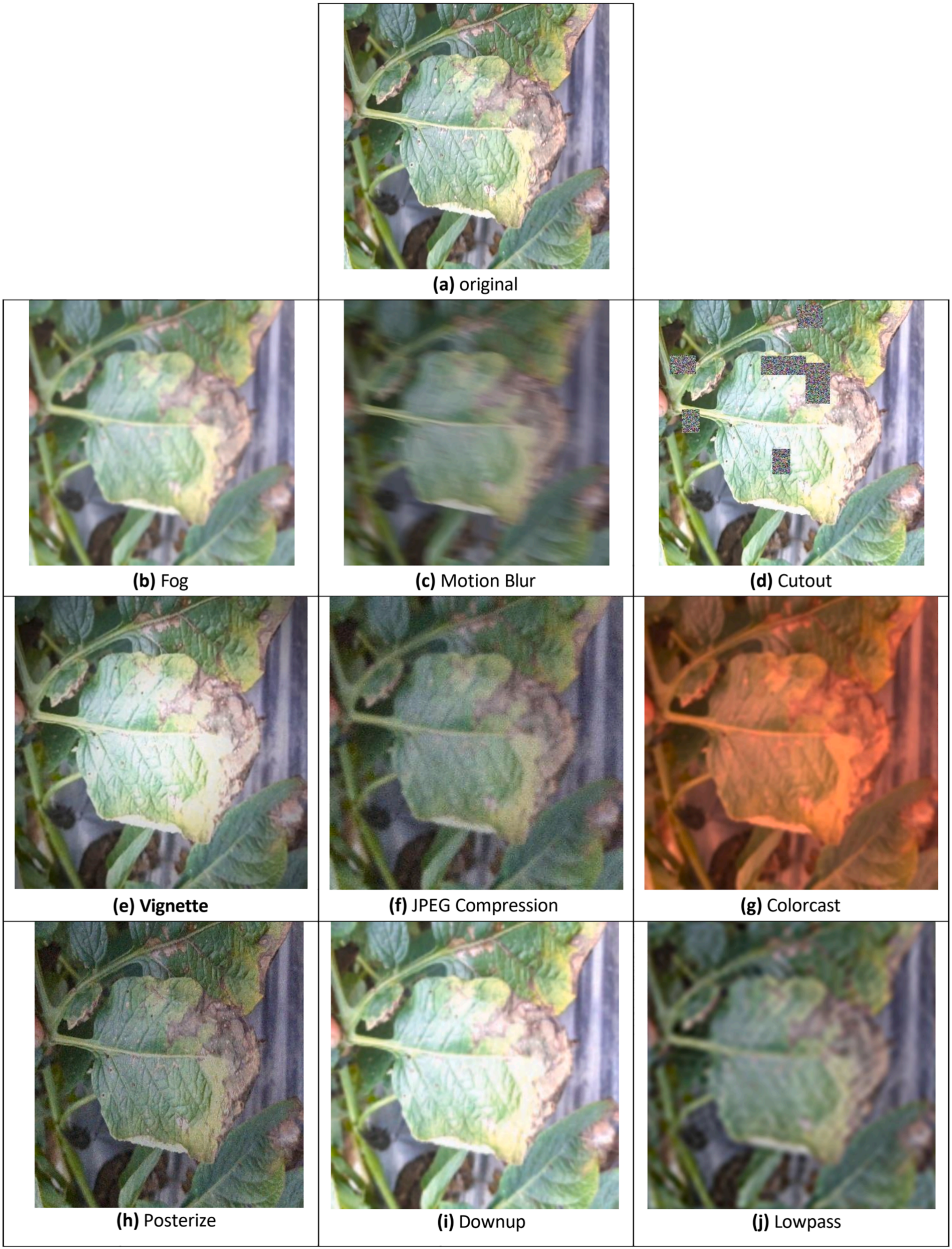
Representative examples of the clean image and the nine corruption families implemented in AgroBench. These image transformations emulate realistic agricultural acquisition degradations and are applied to generate reproducible corrupted validation subsets for robustness evaluation.

In the released implementation, the global modulations are applied first and the family-specific corruption is applied afterward. Annotations are copied unchanged from the clean validation set because the benchmark assumes that the perturbations modify appearance only and do not alter object identity or geometry. This composition rule is fixed throughout the benchmark so that the resulting evaluation subsets remain comparable and reproducible. Representative examples of the clean image and the nine AgroBench corruption families are shown in Fig. 3.

### Corrupted-set generation procedure

For each corruption family f∈F, each family-specific parameter setting $s\in S_{f}$, and each global modulation tuple b∈B, the method generates one corrupted evaluation subset starting from the fixed clean validation set $D_{0}$. The generation procedure modifies image pixels only and preserves the original annotations unchanged. This reflects the assumption that the perturbations affect visual appearance at inference time without changing the identity, location, or geometry of the labeled objects. In the released implementation, labels are copied directly from the clean validation set into the corresponding corrupted-set directory and are reused during evaluation.

The corrupted-set generation workflow is applied independently for each benchmark configuration. First, the clean validation image is read from the reference split. Second, the selected global modulation operator $G_{b}$is applied using the fixed tuple $b=(b_{\text{bright}},b_{\text{blur}},b_{\text{noise}})$. Third, the selected family-specific corruption operator $T_{f,s}$is applied using the chosen corruption family fand parameter setting s. The resulting image is written to the corrupted validation subset corresponding to that benchmark configuration. The annotation file associated with the original clean image is then copied unchanged to the same subset. This procedure is repeated for every image in the clean validation set, producing a complete corrupted evaluation subset $D_{f,s,b}$.

Stochastic elements used during corruption generation, such as occlusion placement, additive noise, and optional hue-related perturbations when enabled, are controlled by a fixed random seed so that the generated corrupted subsets remain identical across runs and across detectors. In contrast, the motion-blur angle is not sampled randomly in the current release, because it is explicitly defined by the family-specific parameter grid. The seed, parameter grids, and corruption configuration are therefore treated as part of the benchmark specification.

In the released implementation, corrupted validation subsets are generated through the script *prepare_valset.py*. One corrupted subset is produced for each combination of corruption family, family-specific parameter setting, and global modulation tuple. As a result, each image in the clean validation set yields exactly one corrupted version for every benchmark configuration enumerated by the method.

### Deterministic evaluation settings

All clean and corrupted evaluations are performed under fixed validation settings so that changes in performance can be attributed to the input perturbations rather than to configuration differences. In the current implementation, the base detection metric is mAP@50, and both clean and corrupted runs use the same Ultralytics validation interface with image size 416, batch size 8, worker count 0, and fixed device selection. Confidence thresholding and non-maximum suppression follow the framework defaults used by this validation call. The clean reference required for ARI is obtained separately under exactly the same validation configuration.

Determinism is enforced at the corruption-generation stage. All stochastic operators use a fixed random seed so that, for any given triple (f,s,b), the corrupted subset $D_{f,s,b}$is regenerated identically across runs. In the current release, corruption generation is deterministic with seed 1004, and hue jitter is disabled unless explicitly stated otherwise. This ensures that model-to-model or run-to-run differences are not caused by different corrupted inputs.

The benchmark definition is stored directly in the released scripts. In particular, the corruption families, family-specific parameter grids, global modulation grids, hue flag, and seed list are defined in *optimize_and_validate_grid.py*. Validation is executed using Ultralytics validation mechanism, and the dataset definition is loaded from the dataset YAML file, with the validation image path rewritten at runtime for each generated corrupted subset.

For each corruption family, the evaluation pipeline exports one CSV trace file containing the corruption parameters and the measured outputs. These outputs constitute the corruption-side audit trail used later for family-level aggregation and ARI computation. Because the same validation settings are used throughout, the method supports direct comparison between clean performance, per-run corrupted performance, per-family retention, and the global ARI score.

### Computation of the Agro-Robustness Index

The Agro-Robustness Index (ARI) is computed from one clean validation reference and a set of corrupted evaluation traces generated under the AgroBench benchmark. The purpose of ARI is to summarize how much of a detector’s clean performance is retained, on average, under the agriculture-calibrated corruption protocol while preserving per-family diagnostic interpretability. In the current implementation, ARI is computed after the corrupted evaluation traces have been generated and exported, using the same detector and the same validation configuration as the clean reference.

The computation proceeds in four stages. First, the detector is evaluated on the clean validation set to obtain the baseline reference score. Second, corrupted performance is collected for every corruption family, every family-specific parameter setting, and every global modulation tuple. Third, corrupted scores are averaged within each corruption family to obtain one family-level mean performance value. Fourth, each family-level mean is normalized by the clean reference to produce a retention ratio, and the global ARI is obtained by averaging these retention ratios across all corruption families. This produces both a scalar robustness summary and a vector of per-family retention components.

#### Clean reference computation

Let Mdenote the evaluated detector and let $D_{0}$denote the clean validation set. The clean reference is computed using the same validation framework and the same evaluation settings used for all corrupted runs. In the present implementation, the base detection metric is mAP@50, and the clean reference is defined as:

$$\phi_{0}\left(M\right)=\phi \left(M;D_{0}\right)$$

where ϕ(·)denotes the selected base detection metric. This clean score serves as the normalization reference for all subsequent ARI calculations. Using the same validation configuration for both clean and corrupted runs is essential, because ARI is intended to measure robustness loss caused by input perturbations rather than by differences in evaluation settings.

#### Family-level aggregation

For each corruption family f∈F, each family-specific parameter setting $s\in S_{f}$, and each global modulation tuple b∈B, the detector is evaluated on the corresponding corrupted subset $D_{f,s,b}$. The corrupted performance for one benchmark run is therefore:

$$\phi_{f,s,b}\left(M\right)=\phi \left(M;D_{f,s,b}\right)$$

Because each family may contain several parameter settings and each setting is combined with all global modulation tuples, the method aggregates these results to obtain one family-level mean performance value. For each corruption family f, the family-level mean is computed as:

$${\overset{‾}{\phi }}_{f}\left(M\right)=\frac{1}{|S_{f}|\;|B|}\sum_{s\in S_{f}}\sum_{b\in B}\phi_{f,s,b}\left(M\right)$$

This aggregation ensures that the score for each family reflects the full released benchmark grid rather than a single severity point or a single modulation condition.

#### Retention ratio and global ARI

ARI is based on normalized retention rather than raw corrupted performance. For each corruption family f, the retention ratio is defined as:

$$\rho_{f}\left(M\right)=\frac{{\overset{‾}{\phi }}_{f}\left(M\right)}{\phi_{0}\left(M\right)+\epsilon }$$

where ϵ>0is a small constant introduced only to avoid numerical instability in degenerate cases. In normal reporting conditions, when the clean reference is strictly positive, ϵcan be set to zero for presentation.

The global ARI score is then computed as the unweighted mean of the family-specific retention ratios:

$$\text{ARI}\left(M\right)=\frac{1}{|F|}\sum_{f\in F}\rho_{f}\left(M\right)$$

This formulation gives ARI a direct interpretation as the average fraction of clean performance retained under the full agriculture-calibrated corruption regime. A higher ARI indicates greater stability under the defined benchmark, whereas the vector of family-level retention ratios identifies the corruption families that contribute most strongly to performance loss.

#### Exported outputs

The released evaluation pipeline exports one CSV trace file per corruption family. Each row corresponds to one evaluated benchmark run and records the corruption family, the parameter dictionary associated with that run, the hue flag, the random seed, the model identifier, and the measured outputs. These outputs include precision, recall, mAP@50, mAP@50:95, inference time, frames per second, GFLOPs, parameter count, and peak GPU memory. Together, these CSV files constitute the corruption-side audit trail used for downstream family-level aggregation and ARI computation.

The final outputs of the method are therefore fourfold:

  1. the clean reference score $\phi_{0}\left(M\right)$,
  2. the per-run corrupted evaluation traces,
  3. the per-family retention ratios $\rho_{f}\left(M\right)$, and
  4. the global ARI score ARI(M).

### Output trace structure and auditability

The AgroBench and ARI workflow was designed so that each stage of corrupted evaluation can be inspected, reproduced, and re-aggregated independently. To support this, the released evaluation pipeline exports **one CSV trace file per corruption family**, with **one row per evaluated benchmark run**. Each row corresponds to one unique combination of corruption family, family-specific parameter setting, global modulation values, hue flag, seed, and evaluated detector. These per-family CSV files constitute the primary audit trail used for downstream robustness aggregation and ARI computation.

In the current implementation, the exported CSV structure is defined directly in the evaluation script *validate_families_grid.py*. The header fields are:

ts,family,params_json,hue,seeds,model,gflops,params,gpu_mem_mb,P,R,mAP50,mAP5095,inf_ms,FPS,cpu_proc_mean_pct,ram_proc_peak_mb,gpu_util_mean_pct

The *params_json* field stores the full parameter dictionary for the evaluated corruption setting, including both the family-specific parameters and the shared global modulation values. In the released script, the global modulation values $b_{\text{bright}}$, $b_{\text{blur}}$, and $b_{\text{noise}}$are embedded in this JSON field rather than stored as separate columns. The *hue* field records whether the optional hue-related perturbation flag was active, and the *seeds* field stores the seed list used for deterministic generation. This design ensures that the full corruption configuration can be reconstructed from the exported trace alone.

The output trace also records both detection metrics and runtime profiling data. The detection fields include precision (*P*), recall (*R*), *mAP50*, and *mAP5095*. The runtime and profiling fields include inference time in milliseconds (*inf_ms*), throughput (*FPS*), best-effort GFLOPs estimation (*gflops*), parameter count (*params*), peak GPU memory (*gpu_mem_mb*), mean process CPU utilization (*cpu_proc_mean_pct*), peak resident RAM usage (*ram_proc_peak_mb*), and mean GPU utilization (*gpu_util_mean_pct*) when available. These outputs allow the user not only to recompute ARI, but also to inspect the computational profile of each evaluated detector under each corruption condition.

Auditability is further strengthened by the deterministic generation logic used in the benchmark. For any fixed corruption family, parameter setting, global modulation configuration, hue flag, and seed, the generated corrupted subset is reproducible across runs. Because the script evaluates all selected models on the same generated corrupted subset before writing the corresponding rows, the resulting trace files can also support fair multi-model comparisons under identical stress conditions. In addition, when two or more models are evaluated, the script saves sample images from the best-mAP50 corrupted subset for each model together with a JSON metadata file describing the selected best configuration Table 3.

**Table 3:** Output trace structure used in the released evaluation script**.**

Field	Description	Purpose
**ts**	Unix timestamp recorded when the evaluation row is written	Identifies when the run was logged
**family**	Corruption family identifier	Distinguishes the evaluated corruption family
**params_json**	JSON-encoded parameter dictionary for the evaluated corruption setting, including family-specific parameters and global modulation values	Records the exact corruption configuration used for the run
**hue**	Binary flag indicating whether hue perturbation was enabled	Records whether the optional hue-based perturbation was active
**seeds**	JSON-encoded list of random seeds used for the run	Ensures deterministic reproducibility of the generated corrupted subset
**model**	Evaluated detector identifier	Links outputs to the tested detector
**gflops**	Best-effort estimate of model computational complexity in GFLOPs	Supports model profiling and efficiency comparison
**params**	Number of model parameters	Supports model profiling
**gpu_mem_mb**	Peak GPU memory allocated during validation, in MB	Supports deployment-oriented analysis
**P**	Detection precision reported by the validation framework	Supports downstream analysis of detection quality
**R**	Detection recall reported by the validation framework	Supports downstream analysis of detection quality
**mAP50**	Mean Average Precision at IoU 0.50	Primary corrupted performance value used in ARI computation
**mAP5095**	Mean Average Precision averaged over IoU thresholds from 0.50 to 0.95	Secondary exported detection metric
**inf_ms**	Inference time reported during validation, in milliseconds	Supports runtime analysis
**FPS**	Throughput computed from inference time	Supports efficiency analysis
**cpu_proc_mean_pct**	Mean CPU utilization of the current process during validation	Supports system-level runtime profiling
**ram_proc_peak_mb**	Peak resident RAM usage of the current process during validation, in MB	Supports system-level memory profiling
**gpu_util_mean_pct**	Mean GPU utilization during validation, when available through NVML	Supports hardware utilization analysis

### Minimal reproduction example

A minimal reproduction of the AgroBench and ARI workflow requires a trained detector, a fixed annotated clean validation set, and the released corruption-generation and evaluation scripts. In the current release, the reproducibility package includes the scripts *prepare_valset.py* and *optimize_and_validate_grid.py*, the implemented benchmark parameter grids, environment specification files, and example commands distributed through the archived software release.

A minimal end-to-end use of the method can be summarized as follows:

1. **Prepare the clean evaluation set.**
2. Define the clean validation subset $D_{0}$and ensure that the corresponding annotation files are available in the detector-compatible format.
3. **Evaluate the clean reference.**
4. Run detector validation on $D_{0}$using the fixed evaluation settings to obtain the clean reference score $\phi_{0}\left(M\right)$.
5. **Generate corrupted validation subsets.**
6. Use the corruption-generation script to enumerate corruption families, family-specific parameter settings, and global modulation tuples, and to generate the corresponding corrupted subsets $D_{f,s,b}$.
7. **Run corrupted evaluations.**
8. Evaluate the detector on all generated corrupted subsets using the same validation configuration used for the clean reference.
9. **Export and inspect trace files.**
10. Verify that one CSV file per corruption family has been produced and that the trace fields contain the expected corruption metadata and evaluation outputs.
11. **Compute ARI.**
12. Combine the clean reference score with the exported corrupted traces, compute the family-level means, derive the per-family retention ratios, and average them across families to obtain the global ARI score.

The expected outputs of a successful minimal reproduction are:

(i) one clean reference metric,
(ii) one exported CSV trace per corruption family,
(iii) one set of family-level aggregated corrupted scores,
(iv) the corresponding per-family retention ratios, and
(v) the global ARI score.

If the method is used on a new dataset, the same reproduction logic applies provided that the clean evaluation set, validation settings, and benchmark grids are clearly documented. The scripts released with the current version were designed so that the corrupted benchmark generation step and the ARI aggregation step can be reproduced separately, which also allows users to inspect the intermediate trace files before computing the final robustness summary.

### Adapting the method to other datasets and detectors

The AgroBench and ARI workflow is intended as a reproducible baseline rather than a fixed universal standard. Although the method is demonstrated here on an agricultural object detection task using YOLOv8n, the same workflow can be reused with other annotated agricultural datasets and with other detector architectures provided that the clean reference and corrupted evaluations are performed under identical validation settings.

#### Adapting to other datasets

When adapting the method to a new dataset, the first requirement is to define a fixed clean evaluation subset that will serve as the reference distribution $D_{0}$. The corruption-generation procedure can then be applied directly to this clean subset while keeping the original annotations unchanged. If the dataset differs substantially in acquisition conditions, crop type, platform, or sensor characteristics, the user may recalibrate the severity grids so that they remain visually plausible for the target context. For example, UAV imagery may require stronger motion or resampling ranges than close-range ground imagery, whereas greenhouse imaging may reduce haze-related effects but introduce stronger specular or illumination artifacts.

To preserve interpretability, any such recalibration should be documented explicitly. A practical reporting strategy is to retain a core subset of corruption families for cross-study comparability and to optionally extend the benchmark with context-specific corruptions when needed. This makes it possible to preserve a common evaluation axis while still tailoring the benchmark to the operating conditions of the target application.

#### Adapting to other detectors

The method is not limited to YOLOv8n. It can be applied to any detector that supports deterministic clean and corrupted evaluation under a fixed metric and validation configuration. In multi-model studies, the same clean subset, corruption protocol, and evaluation settings should be reused across all detectors so that the resulting ARI values remain directly comparable. This is especially important because ARI is designed to measure retained performance relative to each detector’s own clean reference, rather than raw corrupted performance alone.

The original manuscript explicitly positions the present study as a proof of protocol utility rather than as a leaderboard, because only one detector was used in the initial demonstration. A natural extension is therefore to apply the same fixed benchmark to multiple detector families, such as one-stage, two-stage, and transformer-based detectors, and then compare their clean-reference scores, family-level retention vectors, and global ARI values under the same benchmark specification.

#### Practical recommendations for adaptation

When adapting the method, the following practices are recommended:

1. Keep the clean evaluation subset fixed once defined.
2. Preserve the same validation settings for clean and corrupted runs.
3. Document any changes made to severity grids or corruption families.
4. Retain a common subset of corruption families when cross-study comparability is desired.
5. Report both the global ARI value and the per-family retention vector.
6. Avoid tuning training-time augmentations directly to the exact benchmark settings without explicit justification, in order to reduce benchmark leakage.

In this way, the method remains both adaptable and reproducible. It can be specialized to different agricultural imaging scenarios while preserving a transparent benchmark definition and an interpretable robustness summary.

## Method validation

The proposed method was validated on an agricultural object detection task using a YOLOv8n detector and a clean validation subset derived from a 14,150-image potato late blight dataset. The clean reference performance, computed under fixed validation settings, was mAP@50=0.9683. The AgroBench protocol was then applied using nine corruption families, explicit family-specific parameter grids, and twelve global modulation tuples per family-specific setting. Corrupted evaluation traces were generated deterministically and aggregated using the ARI workflow described above.

The resulting validation data show that the method is able to expose robustness behavior that is not visible under clean evaluation alone. Although the detector achieved high clean performance, retention under the corruption benchmark was strongly non-uniform across families. The per-family retention ratios ranged from 0.445 for **Colorcast** to 0.930for **Vignette**, indicating that the same detector can be robust to some field-relevant perturbations while remaining highly vulnerable to others. The global Agro-Robustness Index obtained from these family-level retention values was 0.796, meaning that the detector retained, on average, approximately 79.6%of its clean mAP@50under the full agriculture-calibrated corruption regime.

These validation results support two properties of the method. First, the benchmark and aggregation scheme are sufficiently sensitive to distinguish dominant robustness bottlenecks across corruption families. Second, the retention-based formulation produces a compact global robustness summary while preserving per-family diagnostic interpretability. In the present demonstration, the strongest degradation was observed for chromatic shifts and structured occlusion, whereas vignetting and haze-like degradations produced comparatively smaller average losses. This confirms that the proposed method can be used both for global robustness reporting and for identifying corruption-specific failure modes prior to deployment.

For transparency, the current validation should be interpreted as a protocol demonstration on a single detector rather than as a cross-model benchmark. The released evaluation traces, together with the clean reference score and the archived scripts, allow the same method to be re-applied to additional detectors and datasets under the same benchmark definition.

## Limitations

The present method has several limitations that should be considered when interpreting its outputs.

First, the benchmark is calibrated for agricultural object detection and therefore reflects a domain-specific set of corruption families and parameter grids. While this is one of the strengths of the method for agricultural deployment, it also means that ARI values are conditional on the selected family set F and the corresponding severity grids. Other agricultural contexts, sensor types, and imaging platforms may require re-calibration of the benchmark.

Second, the current validation was performed on a single detector, YOLOv8n. As a result, the article demonstrates the protocol as a robustness profiling method, but does not yet establish cross-model ranking, rank-correlation analysis, or architecture-level comparison under the benchmark. These extensions are natural next steps for future work.

Third, the benchmark relies on synthetic corruptions to approximate real acquisition effects. Although the selected corruption families were chosen to reflect plausible agricultural deployment conditions, synthetic degradations cannot fully reproduce all physical properties of real-world perturbations. The method should therefore be interpreted as a controlled and reproducible stress-testing workflow rather than a complete physical simulator of field conditions.

Fourth, in the current public release, the corrupted benchmark generation and corrupted validation traces are reproduced directly, while the clean reference and final ARI aggregation are obtained as a separate analysis step. This does not prevent reproducibility, but it means that users must combine the exported trace files with a separately computed clean reference when reproducing the final ARI value.

## Ethics statements

This work did not involve human subjects.

This work did not involve animal experiments.

This work did not involve data collected from social media platform.

## Supplementary material *and/or* additional information [OPTIONAL]

The camera-ready software release used to produce the results in this manuscript is archived under the Zenodo version DOI 10.5281/zenodo.18910668 (release *v1.0.1*). The Zenodo concept DOI 10.5281/zenodo.18909108 resolves to the latest available release. The released materials include corruption-generation scripts, evaluation scripts, benchmark configuration details, exported validation traces, environment files, and example commands for reproducing the method.

The dataset used in this study cannot be publicly released due to ownership constraints. Access may be granted for research purposes upon reasonable request and subject to approval by the data owner.

## CRediT authorship contribution statement

**Yassine Zarrouk:** Conceptualization, Methodology, Software, Writing – original draft. **Abdelhak Khallou:** Methodology, Investigation. **Mohammed Bourhaleb:** Supervision, Project administration. **Mohammed Rahmoune:** Investigation, Formal analysis, Data curation. **Khalid Hachami:** Writing – review & editing, Validation, Resources.

## Declaration of competing interest

The authors declare that they have no known competing financial interests or personal relationships that could have appeared to influence the work reported in this paper.

## Data Availability

Data will be made available on request.
